# Synthesis, Spectral Analysis and Preliminary *in Vitro* Evaluation of Some Tetrapyrrolic Complexes with 3d Metal Ions

**DOI:** 10.3390/molecules200915488

**Published:** 2015-08-26

**Authors:** Radu Socoteanu, Gina Manda, Rica Boscencu, Georgiana Vasiliu, Anabela Sousa Oliveira

**Affiliations:** 1 “Ilie Murgulescu” Institute of Physical Chemistry, Romanian Academy, 202 Splaiul Independentei, 77208 Bucharest, Romania; 2 “Victor Babeş” National Institute for Pathology and Biomedical Sciences, 99-101 Splaiul Independenţei, 050096 Bucharest, Romania; E-Mail: gina.manda@gmail.com; 3 Faculty of Pharmacy, “Carol Davila” University of Medicine and Pharmacy, 6 Traian Vuia St., 020956 Bucharest, Romania; E-Mail: georgianavasiliu@yahoo.com; 4 Molecular Physical Chemistry Center and Institute of Nanoscience and Nanotechnology, Instituto Superior Técnico, University of Lisbon, Av. Rovisco Pais, Lisbon 1049-001, Portugal; 5 Interdisciplinary Coordination for Research and Innovation, School of Technology and Management, Polytechnic Institute of Portalegre, Lugar da Abadessa, Apartado 148, Portalegre 7301-901, Portugal

**Keywords:** metalloporphyrins, microwave synthesis, human breast adenocarcinoma MCF-7 cell line, human peripheral mononuclear cells, photodynamic therapy

## Abstract

In this paper, two tetrapyrrolic complexes, Zn(II)-5-(3-hydroxyphenyl)-10,15,20-tris-(4-acetoxy-3-methoxyphenyl)porphyrin and Cu(II)-5-(3-hydroxyphenyl)-10,15,20-tris-(4-acetoxy-3-methoxyphenyl)porphyrin were synthesized, and characterized from a spectral and biological point of view. The study provided data concerning the behavior of identical external substituents *vs.* two different core insertions. Some of the properties of the proposed tetrapyrrolic structures were highlighted, having photodynamic therapy of cancer as a targeted biomedical application. Elemental analysis, NMR, FTIR and UV-Vis data in various solvents were provided. A preliminary *in vitro* study on normal and cancer cultured cells was carried out for biocompatibility assessment in dark conditions. The preliminary *in vitro* study performed on human peripheral mononuclear cells exposed to tetrapyrrolic compounds (2 µM) showed that the proposed compounds had a convenient cytotoxic profile on human normal peripheral blood mononuclear cells under dark conditions. Meanwhile, the investigated compounds reduced the number of metabolically active breast tumor MCF-7 cells, with the exception of Zn(II) complex-containing a symmetrical ligand. Accordingly, preliminary *in vitro* data suggest that the proposed tetrapyrrolic compounds are good candidates for PDT, as they limit tumor expansion even under dark conditions, whilst sparing normal cells.

## 1. Introduction

Tetrapyrrolic compounds, especially porphyrins and metalloporphyrins, are still a spearhead in the studies related to diagnosis and photodynamic therapy (PDT) of malignant tumours [1,2,3,4,5,6,7,8,9]. The therapeutic effect of tetrapyrrolic compounds results from their ability to accumulate preferentially in the malignant tissue and to generate, following light-mediated activation in the presence of molecular oxygen, reactive oxygen species responsible for cell death [10,11,12]. The use of porphyrinic compounds as fluorescent markers in the detection of various cancer types is based on their photophysical characteristics, such as emissions in the spectral range 600–800 nm, large Stokes shift and high fluorescence lifetime [2,3].

In diagnostic and therapeutic applications the efficiency of tetrapyrrolic compounds depends on their structural and physical-chemical characteristics [13]. High absorption coefficients in the 600–680 nm spectral range, high singlet oxygen generation quantum yield, acceptable solubility in biologic fluids, selectivity for diseased tissues and superior uptake values, photostability and lack of toxicity in the absence of light excitation are the most important parameters that dictate the efficiency of porphyrins as photosensitizers [1,2,3].

Current mainstream research approaches include more and more complex long-chain studies of both porphyrins and metalloporphyrins configured as third generation photosensitizers [14,15]. The quest for optimal or at least most improved structures, remains active, despite the already clinical approved porphyrin-base drugs [16]. Among metalloporphyrins, some forms with Zn(II) have been recently studied as starting points for future efficient photodynamic agents, mostly with a focus on asymmetrically substituted bases; meanwhile, copper porphyrins are less present in this type of studies, for instance as compounds involved in the side-effects of phototherapy [17,18,19,20].

**Figure 1:**
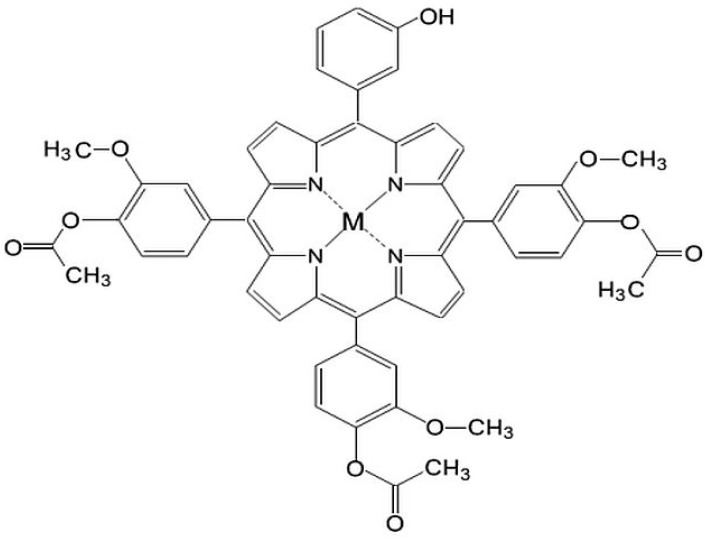
General structures of M(II)-5-(3-hydroxyphenyl)-10,15,20-tris-(4-acetoxy-3-methoxyphenyl)porphyrin, M = Cu, Zn.

Taking into account that localization at a subcellular level of the tetrapyrrolic structure is directly influenced by the polarity of the molecule and by the type of metallic ion [21,22], our research has been focused on obtaining porphyrins with various degrees of hydrophobic/hydrophilic substitutions that favor their uptake by cellular targets [23,24,25,26,27,28,29,30]. As a continuation of our research, in this study we describe the synthesis of some new asymmetrical tetrapyrrolic complexes, Zn(II)-5-(3-hydroxyphenyl)-10,15,20-tris-(4-acetoxy-3-methoxyphenyl)porphyrin (**P1**) and Cu(II)-5-(3-hydroxyphenyl)-10,15,20-tris-(4-acetoxy-3-methoxyphenyl)porphyrin (**P2**) (Figure 1), their spectral properties and a preliminary *in vitro* evaluation focused on their dark toxicity in the context of their potential use in the diagnosis and PDT of cancer.

## 2. Results and Discussion

### 2.1. Chemistry

The syntheses presented in this study have been successfully repeated several times with identical results. The dry media synthesis procedure was performed in three stages: irradiation followed by cooling and with UV-Vis analytical monitoring of samples. After synthesis and purification, the porphyrinic complexes were characterized by elemental analysis, FTIR, ^1^H- and ^13^C-NMR and UV-Vis spectrometry.

The infrared spectral data obtained for the synthesized metalloporphyrins revealed the presence of typical vibration modes of both the tetrapyrrolic macrocycles and phenyl substituents. This is generally in agreement with results previously reported for similar porphyrinic structures [25,26,27,28,29,30]. The large band registered at about 3410 cm^−1^ can be assigned to the O–H stretching vibration of the –OH functional group, near the typical 3423 cm^−1^ of the N–H vibration. In the higher wavenumber region at ~2920 cm^−1^ a medium band was registered, corresponding to C–H vibrations of the phenyl groups. Around 2850 cm^−1^ the band corresponding to C–H vibration frequencies of the –O–CH_3_ group was detected. For both synthesized metalloporphyrins the IR signals at 1709 cm^−1^ can be assigned to the C=O stretching vibration, while the absorption band evidenced at 1104 cm^−1^ was attributed to the C–O bond vibrations. In the infrared spectra of **P1** and **P2**, the bands corresponding the C=N and C–N stretching vibrations were highlighted in the spectral range of 1490–1510 cm^−1^ and 1600 cm^−1^, respectively. Several signals belonging to the pyrrole ring are also present at 975 cm^−1^ for δ_C-H_ and 798 cm^−1^ for γ_C-N_. The general shape of the spectra remains similar for both complexes even below 1600 cm^−1^; in this region several medium intensity signals attributed to C–C vibrations as 1397, 1349, 1326 cm^−1^ or γ_C-C_ at 880 and 846 cm^−1^ are present.

#### Absorption and Emission Spectra

The complexes were studied by UV-Vis spectroscopy to confirm their structure and to establish their profiles in environments with different polarities. Due to solvent dependence of the position of the Soret band and all the other bands, measurements were concentrated around organic solvents with impact in biomedical studies. Due to the presence of –OH groups at the periphery of the tetrapyrrolic complex, the solubility was increased, but remained an issue for systems containing water.

**Figure 2:**
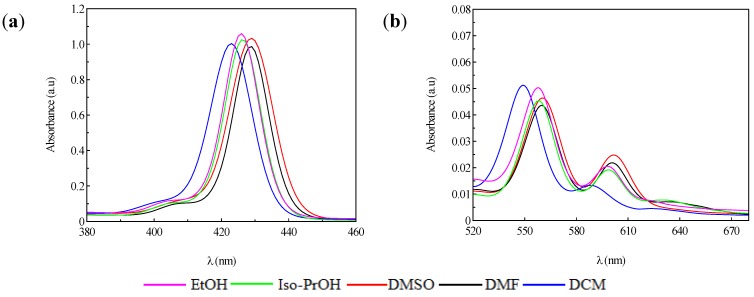
Absorption spectra of **P1** in different solvents; (**a**) Soret bands; (**b**) Q bands.

Parameters of the solvents from the polarity to the donor-acceptor process often cause significant changes in the band energy values leading to shifts in the spectral profiles. As expected, in our particular case the Soret band shifted in a narrow range of 4 nm for **P1** but doubled in the case of **P2**. The relative intensities were sensitive to the nature of the solvent, but within narrow limits as can be seen in Table 1 and Figure 2.

**Table 1:** Absorption and emission data of the porphyrinic complexes in various solvents (*c* = 2.5 × 10^−6^ M, λ_ex_ = 420 nm).

Solvent	Absorption λ_max_ (nm) [lgε (L·mol^−1^·cm^−1^)]	Emission λ_max_ (nm)
	Soret Band B Q_y_ (0,0) Q_x_(1,0)	Q(0,0) Q(0,1)
Zn(II)-5-(3-hydroxyphenyl)-10,15,20-tris-(4-acetoxy-3-methoxyphenyl)porphyrin		
EtOH	426 [5.63] 558 [4.30] 598 [3.92]	605 653
*Iso*-PrOH	426 [5.61] 559 [4.21] 599 [3.90]	605 652
DCM	423 [5.60] 549 [4.30] 588 [3.71]	603 650
DMF	428 [5.58] 561 [4.24] 600 [3.94]	608 656
DMSO	430 [5.61] 560 [4.26] 602 [4.00]	609 656
**Cu(II)-5-(3-hydroxyphenyl)-10,15,20-tris-(4-acetoxy-3-methoxyphenyl)porphyrin**		
EtOH	413 [5.52] 537 [4.28]	- -
*Iso*-PrOH	414 [5.58] 538 [4.36]	- -
DCM	416 [5.50] 540 [4.14]	- -
DMF	420 [5.52] 542 [4.20]	- -
DMSO	421 [5.51] 544 [4.30]	- -

EtOH = ethanol, *Iso*-PrOH = isopropyl alcohol, DCM = dichloromethane, DMF = dimethylformamide, DMSO = dimethyl sulfoxide.

The insertion of an ionic metal in the porphyrinic core led to a decrease of the vibrational modes evidenced by the reduced number of Q bands. In this context, **P1** displayed two Q bands, Qy(0,0) and Qx(1,0), respectively. Meanwhile, in the case of **P2**, the spectrum contained only one Q band.

**Figure 3:**
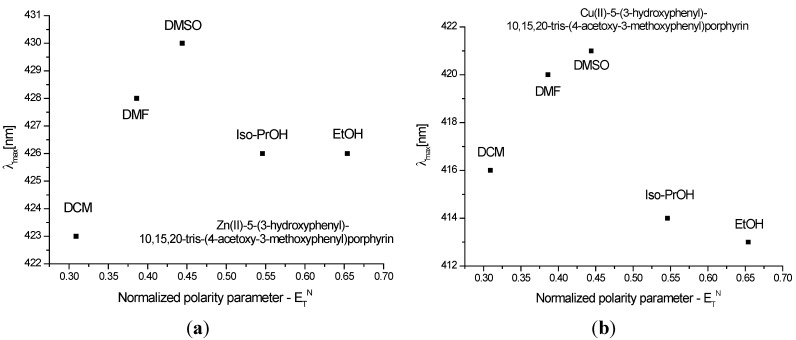
Reichardt’s normalized polarity parameter *vs.* wavelengths maxima of the Soret bands for (**a**) **P1** and (**b**) **P2**.

**Figure 4:**
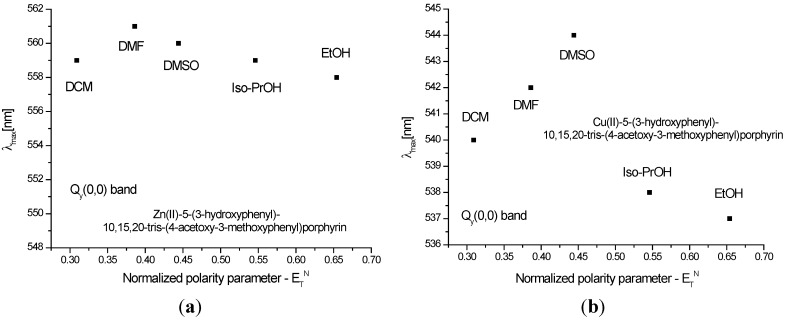
Reichardt’s normalized polarity parameter *vs.* wavelength maxima of the Qy(0,0) bands for (**a**) **P1** and (**b**) **P2**.

Figure 3 and Figure 4 show the quenching influence of the more polar solvents both on Soret band and on the Q bands. The general tendencies are the same for **P1** and **P2**, despite the small differences between these compounds, given in part by the different complexation power of the two metals. The absence of **P2** emission is in agreement with data reported in the literature [31,32,33]. **P1** on the other hand had two distinctive signals. The differences in terms of peak maximum values remained constant and independent of the solvent. Dichloromethane proved to have the most significant hypsochromic influence, suggesting that less polar solvents seem to be more suitable for further biological tests.

### 2.2. In Vitro Dark Cytotoxicity Study

Tetrapyrroles for biomedical applications with suitable physico-chemical and photophysical properties, are generally selected in a first stage of development based on their low dark toxicity. This can be assessed by *in vitro* studies on normal and diseased cells. To this end we studied the effects of the proposed porphyrinic compounds on human cancer cells (the breast human carcinoma MCF-7 cell line) and on human normal peripheral blood mononuclear cells (PBMC). The *in vitro* study focused on the viability and/or proliferation of the mentioned cells, assessed by the MTS reduction test that provides information regarding the number of metabolically active cells in a culture. We investigated the ligand 5-(3-hydroxyphenyl)-10,15,20-tris-(4-acetoxy-3-methoxyphenyl)-21,23-*H*-porphyrin (**P**) [28] and its complexes with Zn(II) and Cu(II) Zn(II)-5-(3-hydroxyphenyl)-10,15,20-tris-(4-acetoxy-3-methoxyphenyl)porphyrin (**P1**) and Cu(II)-5-(3-hydroxyphenyl)-10,15,20-tris-(4-acetoxy-3-methoxy-phenyl)porphyrin (**P2**). The role of the asymmetry element that was introduced in the structure of the porphyrinic compound was studied in relation to the cytotoxicity profile. Accordingly, the series of symmetric porphyrinic compounds was tested in parallel with the asymmetric ones: 5,10,15,20-*meso*-tetrakis-(4-acetoxy-3-methoxyphenyl)-21,23-*H*-porphyrin (**P3**), Zn(II)-5,10,15,20-*meso*-tetrakis-(4-acetoxy-3-methoxyphenyl)porphyrin (**P4**), Cu(II)-5,10,15,20-*meso*-tetrakis-(4-acetoxy-3-methoxy-phenyl)porphyrin (**P5**) [29]. The compounds dissolved in DMSO were tested *in vitro* at the concentration of 2 μM, corresponding to a 0.12% final concentration of DMSO in the cell cultures.

#### The Effect of Porphyrinic Compounds on Human Tumor MCF-7 Cells

**Figure 5:**
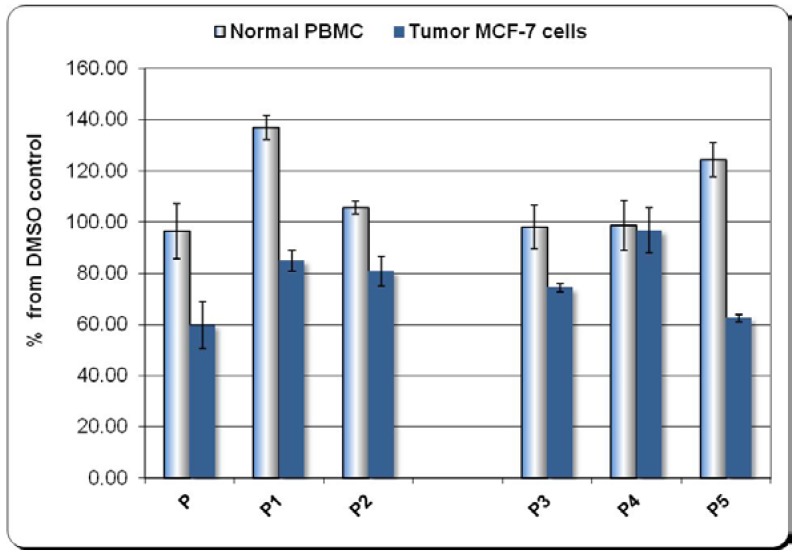
The effect exerted *in vitro* by the groups of compounds **P**, **P1**, **P2** and **P3**, **P4**, **P5** on the number of metabolically active cells in culture, either on normal human peripheral mononuclear cells (PBMC), or on human breast carcinoma MCF-7 cells. Evaluation by the MTS reduction test. Results were presented as percentage of MTS reduction in presence of compounds from the corresponding DMSO-induced MTS reduction (mean ± SEM for three independent experiments).

Results presented in Figure 5 show that all the tested compounds at 2 µM concentration did not affect the viability of normal PBMC (**P**, **P2**, **P3**, **P4**) or even tended increase the MTS reduction (**P1**, **P5**).

Meanwhile, most of the investigated compounds reduced the number of metabolically active tumor cells (**P**, **P1**, **P2**, and **P3**, **P5**). This cytotoxic effect was more pronounced in the case of compounds **P** and **P5**. In turn, the Zn(II)-containing complex (**P4**) exerted no major effect on tumor cells. Compounds **P** and **P5** proved to be promising candidates for cancer therapy by drastically inhibiting the growth of tumor cells, whilst having no cytotoxic action on normal PBMC. We emphasize that the Zn(II)-containing compound (**P4**) had almost no impact on normal and cancer cells

## 3. Experimental Section

### 3.1. General Information

Commercially available chemicals and solvents from Sigma-Aldrich (St. Louis, MO, USA) and Merck (Whitehouse Station, NJ, USA) were used for chemical synthesis. For the microwave-assisted synthesis a MWG775H type temperature-controlled microwave oven (Clatronic, Kempen, Germany) was used. The elemental analysis of C, H and N was performed with an automatic 1108 analyzer (Carlo Erba, Milan, Italy). IR spectra were recorded with a FT-IR Tensor 27 spectrophotometer (Bruker, Fremont, CA, USA). The UV-Vis spectra of the porphyrinic complexes were recorded with a Lambda 35 spectrophotometer (Perkin-Elmer, Waltham, MA, USA) and fluorescence spectra were recorded with a FP 6500 spectrofluorimeter (Jasco, Tokyo, Japan). The metalloporphyrin solutions in different organic solvents (ethanol, isopropyl alcohol, dimethylformamide, dichloromethane, dimethyl sulfoxide) were freshly prepared in the spectrally pure solvents at the concentration 2.5 × 10^−6^ M and kept in dark until the measurement to prevent photodegradation. The NMR spectra of the porphyrinic complex were recorded with a 400 MHz NMR spectrometer (Bruker).

### 3.2. Synthesis of Tetrapyrrolic Complexes

#### 3.2.1. Synthesis of Zn(II)-5-(3-hydroxyphenyl)-10,15,20-tris-(4-acetoxy-3-methoxyphenyl)porphyrin (**P1**)

**Scheme 1:**
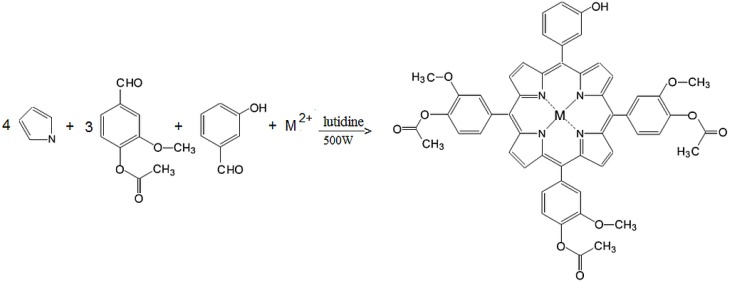
General scheme for synthesis of **P1** and **P2** complexes.

A mixture of 3-hydroxybenzaldehyde (1.22 g, 0.01 mol), methyl 4-formyl benzoate (5.82 g, 0.03 mol), freshly distilled pyrrole (2.76 mL, 0.04 mol), anhydrous zinc chloride (1.36 g, 0.01 mol) and 2.5–3 g of silica gel 60 (200–500 μm, 35–70 mesh) in the presence of 2,6-dimethylpyridine (1 mL) was irradiated in a Pyrex bottle in a microwave oven set at 180 °C, 500 W for 10 min (Scheme 1). The complexation reaction was monitored using UV-Vis spectroscopy and TLC. Extraction of samples was performed after every 2 min of irradiation. TLC test of the final product of reaction revealed the presence of a six metalloporphyrin isomers (A4, A3B, A2B2 (*cis* and *trans*), AB3 and B4-type) with high content of A3B isomer. The crude product was first dissolved in dichloromethane/diethyl ether 25:1 (*v*/*v*), filtered and purified on a chromatography column by repeated elution, using silica gel (100–200 mesh size) as stationary phase and dichloromethane/diethyl ether 25:1 (*v*/*v*) as eluent. The solution of the Zn porphyrinic compound was concentrated by simple distillation, the obtained violet crystals were dried at 100 °C for 12 h. The yield was about 28%. Elemental analysis for C_53_H_40_N_4_O_10_Zn: calculated (found): C 66.45(66.35), H 4.18(4.10), N 5.85 (5.69). ^1^H-NMR (CDCl_3_), δ_H_ ppm: 3.90 (s, 9H), 4.0 (s, 9H), 5.98 (s, 1H), 7.15 (d, *J* = 8.5 Hz, 3H), 7.25 (s, 3H), 7.45 (d, *J* = 8.5 Hz, 3H), 7.70 (m, 1H), 7.75 (t, 1H), 7.80 (d, *J* = 8.0 Hz, 1H), 7.0 (s, 1H), 8.80 (d, *J* = 4.70 Hz, 6H), 8.92 (d, *J* = 4.72 Hz, 2H). ^13^C-NMR (CDCl_3_), δ_C_ ppm: 53.6, 76.7, 81.0, 115.0, 118.0, 120.0, 121.8, 122.0, 127.7, 128.0, 130.0, 132.3, 134.8, 139.0, 146.8, 150.3. IR (cm^−^^1^): 3408, 2922, 2851, 1709, 1601, 1496, 1434, 1267, 1104, 995, 791, 759. UV-Vis (CH_2_Cl_2_) λ_max_ (nm): 423, 550, 588.

#### 3.2.2. Synthesis of Cu(II)-5-(3-hydroxyphenyl)-10,15,20-tris-(4-acetoxy-3-methoxyphenyl)porphyrin (**P2**)

The synthesis of **P2** was performed by a similar method to that of **P1** (Scheme 1), by microwave irradiation at 500 W in the presence of 2,6-dimethylpyridine, of a mixture of 3-hydroxybenzaldehyde (1.22 g, 0.01 mol), methyl 4-formyl benzoate (5.82 g, 0.03 mol), pyrrole (2.76 mL, 0.04 mol) and anhydrous CuCl_2_ (1.34 g, 0.01 mol). The presence of the porphyrinic complex in the reaction mixture was confirmed by monitoring the Q bands in the visible spectra recorded for extracted samples after every 2 min of irradiation. After cooling, the reaction product was dissolved in dichloromethane/diethyl ether 25:1 (*v*/*v*) and then filtered. The solvent was removed under vacuum and the solid product was chromatographically separated on silica gel (100–200 mesh size) with dichloromethane/diethyl ether as eluent. The solution of the Cu porphyrinic complex was concentrated by simple distillation and the obtained dark red crystals were dried at 100 °C for 12 h. The obtained yield was 33%. Elemental analysis for C_53_H_40_N_4_O_10_Cu: calculated (found) C 66.56 (66.42), H 4.18 (4.10), N 5.86 (5.72). ^1^H-NMR (CDCl_3_), δ_H_ ppm: 3.90 (s, 9H), 4.15 (s, 9H), 6.10 (s, 1H), 7.18 (d, *J* = 8.5 Hz, 3H), 7.25 (s, 3H), 7.48 (d, *J* = 8.5 Hz, 3H), 7.72 (m, 1H), 7.77 (t, 1H), 7.81 (d, *J* = 8.0 Hz, 1H), 7.0 (s, 1H), 8.78 (d, *J* = 4.70 Hz, 6H), 8.90 (d, *J* = 4.72 Hz, 2H).^13^C-NMR (CDCl_3_), δ_C_ ppm: 53.3, 77.0, 81.0, 115.0, 119.0, 120.0, 121.8, 122.3, 128.0, 128.2, 130.0, 132.3, 134.5, 139.0, 146.8, 152.2. IR (cm^−1^): 3413, 2919, 2850, 1709, 1603, 1507, 1437, 1277, 1104, 995, 798, 722. UV-Vis (in CH_2_Cl_2_) λ_max_ (nm): 416, 540. 

### 3.3. In Vitro Dark Cytotoxicity Tests

#### 3.3.1. Preparation of Tetrapyrrolic Compounds for the *in Vitro* Study

Tetrapyrrolic compounds were dissolved initially in DMSO and were further diluted in culture medium before the experiments to obtain working concentrations of 2 μM. The final DMSO concentration in cell cultures was 0.12%.

#### 3.3.2. Cells

The human breast carcinoma MCF-7 cell line was purchased from ATCC (ATCC^®^ HTB-22™) and was cultivated in DMEM (Biochrome, Berlin, Germany) culture medium supplemented with 10% fetal bovine serum (FBS, Biochrome) and antibiotic-antimycotic solution (Sigma-Aldrich). Passage of cells was performed at 48 h by trypsinization of adherent MCF-7 cells using 0.25%/0.02% Trypsin/EDTA (Gibco, Life Technologies, Carlsbad, CA, USA). For experiments, MCF-7 cells were plated in 96 well culture plates, at a density of 40,000 cells/cm^2^. Human peripheral mononuclear cells (PBMC) were isolated from blood samples of 6 healthy volunteers, based on their informed consent. Briefly, 3 mL blood collected in Li-heparin coated vacutainers was layered on Biocoll solution (Biochrome), and was centrifuged for 20 min at 2000 rpm, at room temperature. Mononuclear cells were collected and were washed twice in RPMI 1640 culture medium (Biochrome) by centrifugation for 10 min at 1500 rpm, 4 °C. Cells were finally suspended in RPMI 1640 medium supplemented with 10% FBS (Biochrome) and antibiotic-antimycotic solution (Sigma-Aldrich). Cells were counted in a Burker-Turk hemocytometer. Cellular viability was assessed by the trypan blue exclusion test. Only cell suspensions with viability >95% were used for experiments.

#### 3.3.3. Cell Cultures

For experiments, MCF-7 cells were plated in 96 well culture plates, 7000 cells/well (21,000 cell/cm^2^), and were cultivated for 24 h to reach adherence. PBMC isolated from 6 healthy volunteers were mixed for obtaining activated PBMC by mixed lymphocyte reaction. PBMC were thereafter plated in 96 well culture plates, 10^5^ cells/well. Cells were treated with the test substances (2 μM concentration). Non-treated cells and cells treated with the solvent (DMSO) were used as controls. Samples containing culture medium and dilutions of the test substances were used for background assessment. Final sample volume in experiments was 100 μL. All samples were plated in triplicate. Cell cultivation was performed at 37 °C, in 5% CO_2_ atmosphere.

#### 3.3.4. The MTS Reduction Test

The MTS reduction test was used for assessing the number of metabolically active cells in culture, using the CellTiter 96^®^ AQueous One Solution Cell Proliferation Assay (Promega, Madison, WI, USA). Cells were cultivated as described above, and treated with test substances for 48 h. At the end of the treatment time, 20 μL of the kit reagent were added to each well of the culture plate. Cells were further incubated for 3 h at 37 °C, in 5% CO_2_ atmosphere. The optical density (OD) of samples was measured at 490 nm against 620 nm reference, using a Sunrise ELISA reader (Tecan, Männedorf, Schweiz). Final OD in cellular samples was calculated by subtracting the OD of corresponding background samples. Data were processed as percentage of test sample OD relative to DMSO-corresponding OD, and were presented as mean ± standard error of the mean (SEM) for independent experiments.

## 4. Conclusions

The spectral data confirmed the expected general profiles of the studied tetrapyrrolic complexes, and placed them in suitable position for the next step in our quest to identify convenient photosensitizers. The preliminary *in vitro* study indicated that the new porphyrinic complexes did not display dark condition cytotoxic effects on normal PBMC, and generally reduced the number of metabolically active tumor MCF-7 cells. Taking into account that an appropriate PS candidate for efficient and safe PDT in tumors should minimally affect normal and diseased cells under dark conditions, the Zn(II)-containing compounds proved to be more convenient for potential biomedical use than Cu(II)-containing ones.
